# Serotypes, antibiotic susceptibility and whole-genome characterization of Streptococcus pneumoniae in Sichuan Province, China in 2023

**DOI:** 10.1099/jmm.0.002107

**Published:** 2025-12-18

**Authors:** Shu Huang, Laihong Shen, Rongmei Yuan, Hongyu Liao, Wenbo Li, Guo Chen, Tianrong Li, Linzi Zeng

**Affiliations:** 1 Sichuan Center for Disease Control and Prevention, Chengdu 610041, PR China; 2 Panzhihua Center for Disease Control and Prevention, Panzhihua 617099, PR China; 3 Mianyang Center for Disease Control and Prevention, Mianyang 621099, PR China; 4 Nanchong Center for Disease Control and Prevention, Nanchong 637001, PR China

**Keywords:** antibiotic susceptibility, molecular characterization, serotype, *Streptococcus pneumoniae*, whole-genome sequencing

## Abstract

**Introduction**. *Streptococcus pneumoniae* is a leading cause of pneumonia, meningitis and other invasive diseases. The increasing prevalence of antibiotic resistance in *S. pneumoniae* has become a major public health concern, complicating clinical treatment and highlighting the need for continuous surveillance.

**Gap Statement**. Although previous studies have reported the antimicrobial resistance, serotype distribution and molecular characteristics of *S. pneumoniae* in some regions of China, systematic and whole-genome sequencing-based epidemiological data for Sichuan Province remain limited. Currently, there is a lack of comprehensive understanding regarding the predominant serotypes, resistance profiles, virulence gene carriage and their relationship with global clonal complexes in this region. In particular, data supporting the assessment of vaccine-covered serotypes and the transmission risk of multidrug-resistant strains are insufficient.

**Aim**. This study aimed to investigate the prevalence, serotype distribution, antimicrobial resistance and molecular characteristics of *S. pneumoniae* isolates in Sichuan Province, providing genomic evidence to support rational antibiotic use and optimize immunization strategies.

**Methodology**. A total of 105 clinical *S. pneumoniae* strains were collected in 2023 through the China Pathogen Identification Net. The serotypes, molecular types and antibiotic resistance of the strains were determined by whole-genome sequencing, sequence analysis and antimicrobial susceptibility test.

**Results**. The leading serotypes were 19F (34.29%), 19A (10.48%), 3(7.62%) and 6E (7.62%). The dominant sequence types (STs) were ST271 (30.48%) and ST320 (10.48%), with CC271 and GPSC1 being the predominant groups. Vaccine coverage rates were 45.71% for PCV7/PCV10, 64.76% for PCV13, 69.52% for PCV20 and 70.48% for PPV23. All isolates were susceptible to linezolid and vancomycin, but high resistance was observed to erythromycin (92.38%), clindamycin (82.86%), tetracycline (86.87%) and trimethoprim/sulfamethoxazole (60.95%). The multidrug resistance (MDR) rate was 85.71%. Nine resistance genes were identified, with *erm(B*) and *tet(M*) being the most prevalent.

**Conclusion**. Our study provides reliable information, including the prevalence, molecular characterization and antimicrobial resistance of *S. pneumoniae* isolates in Sichuan Province of China in 2023. The high MDR rate and predominance of vaccine-covered serotypes highlight the urgent need for enhanced surveillance, rational antibiotic use and broader implementation of pneumococcal vaccines, including the potential introduction of PCV20.

**Impact Statement**

Our study provides valuable insights into the serotypes, molecular characteristics and antibiotic susceptibility of *Streptococcus pneumoniae* strains in Sichuan Province. The most prevalent serotypes in this study were 19F, 19A, 3 and 6E; the most prevalent Global Pneumococcal Sequencing Clusters (GPSCs) were GPSC1, GPSC12 and GPSC23; the most prevalent sequence types (STs) were ST271 and ST320; and the most prevalent clonal complex (CC) was CC271. All strains were sensitive to vancomycin and linezolid, but highly resistant to erythromycin, clindamycin, tetracycline and trimethoprim/sulfamethoxazole, with a concerning multidrug resistance of up to 85.71%. These data highlight the importance of appropriate use of antibiotics and emphasize the necessity of monitoring pneumococcal epidemiology. In our study, PCV13 covered 64.76% of *S. pneumoniae* isolates, PCV20 covered 69.52% and PPV23 covered 70.48%. Given that only PCV13 and PPV23 are currently licensed in China and the persistently high rate of antibiotic non-susceptibility, efforts should be made to consolidate the implementation of PCV13 and PPV23 while actively facilitating the clinical introduction of PCV20. This combined strategy will improve the prevention and control of pneumococcal infections in Sichuan Province. Continuous monitoring of the molecular characteristics and antibiotic resistance of *S. pneumoniae* is crucial for controlling and preventing pneumococcal infections.

## Data Summary

The authors confirm that all supporting data and protocols have been provided within the article or through supplementary data files.

## Introduction

*Streptococcus pneumoniae* is a Gram-positive diplococcus, with humans as its sole host. It often colonizes in the nasopharynx of the human body and is a common opportunistic pathogen [1]. When the body’s immune system is weakened, *S. pneumoniae* can cause diseases such as pneumonia, bacteraemia, otitis media and meningitis. It has become the main pathogenic bacterium of community-acquired pneumonia [2]. A global study on causes of death has also proved that more than half of the deaths caused by lower respiratory tract infections are attributed to infections with *S. pneumoniae* [3]. Besides, *S. pneumoniae* is one of the main pathogens causing pneumonia and meningitis in children under 5 years old in China [4].

Currently, there are over 100 serotypes of *S. pneumoniae* [5]. The prevalence of *S. pneumoniae* varies in different regions of China. For example, the serotypes of 19F, 23F, 6B, 14, 15B and 22F are common in northern regions, such as Beijing and Tianjin [6]. In southern regions, such as Guangdong and Shanghai, in addition to the above serotypes, serotypes 1, 5 and 7F also account for a certain proportion [7]. Although pneumococcal vaccines have a good protective effect against vaccine serotypes of invasive pneumococcal disease [8,9], the current ‘serotype replacement’ has seriously undermined the protective effect of the vaccine [10,11]. Therefore, a long-term and continuous monitoring of pneumococcus is the basis for the prevention and control of pneumococcal disease, risk assessment, vaccine development and evaluation of vaccine effectiveness. With the abuse of antibiotics, the issue of antibiotic resistance in *S. pneumoniae* cannot be ignored. According to the statistics of the Global Antimicrobial Surveillance System of the World Health Organization (WHO), *S. pneumoniae* is one of the four most commonly reported bacterial strains for antibiotic resistance [12]. According to the Bacterial Priority Pathogens List released by WHO in 2024, *S. pneumoniae* is classified as a moderate priority pathogen due to its macrolide resistance [13] (https://www.who.int/publications/i/item/9789240093461). A study published by the Global Burden of Disease Working Group also shows that *S. pneumoniae* is one of the six major pathogens closely related to bacterial resistance [14]. The antibiotic resistance of *S. pneumoniae* in China is serious. Studies have shown that the resistance rate of *S. pneumoniae* to erythromycin in Chinese children is the highest, reaching 96.4%, followed by tetracycline and sulfamethoxazole [6]. Our study aims to explore the molecular characteristics and antibiotic resistance of *S. pneumoniae* through whole-genome sequencing and antibiotic susceptibility testing, providing a relevant reference for the epidemic prevention and control of *S. pneumoniae* in Sichuan Province of China.

## Methods

### Isolation and identification of *S. pneumoniae* strains

Through the China Pathogen Identification Net, we collected 105 clinical *S. pneumoniae* strains in 2023 from ten cities: Chengdu (8), Dazhou (4), Deyang (3), Guangyuan (7), Luzhou (15), Mianyang (16), Nanchong (16), Neijiang (13), Panzhihua (19) and Ziyang (4). The cultured samples were blood (4 strains), cerebrospinal fluid (1 strain) and respiratory tract (100 strains), including sputum (96 strains) and bronchoalveolar lavage fluid (4 strains). The gender information of 1 patient was missing, and the remainder included 65 males and 39 females. Of these, 73 patients (69.52%) were ≤18-year-old children, 10 patients were 18~65 years old and 22 patients (20.95%) were older than 65 years old (Table S1, available in the online Supplementary Material). According to the ‘National Guide to Clinical Laboratory Procedures’, the strains were inoculated onto Colombian blood plates and cultured at 37 ℃ in a 5% CO_2_-enriched atmosphere for 18–20 h. After cultivation, the identification was confirmed by an automatic matrix-assisted laser desorption/ionization time-of-flight mass spectrometry EXS3000 (Zybio, Chongqing, China). The *S. pneumoniae* isolates are stored in skim-milk tryptone glucose glycerin (STGG) at −80 °C for subsequent analysis.

### Antimicrobial susceptibility test

Subcultured *S. pneumoniae* strains on the Colombian blood plates were used for antibiotic susceptibility test by the Customized AST plate CHNSTRF from Thermo Fisher Scientific (Waltham, MA, USA), using the following agents: penicillin (PEN), cefepime (FEP), cefotaxime (CTX), amoxicillin (AMX), erythromycin (ERY), meropenem (MEM), vancomycin (VAN), clindamycin (CLI), chloramphenicol (CHL), tetracycline (TCY), moxifloxacin (MFX), levofloxacin (LVX), trimethoprim/sulfamethoxazole (SXT) and linezolid (LNZ). The broth medium containing split horse blood CAMHB-LHB also comes from Thermo Fisher Scientific. After culturing for 18–20 h, the results were read using Thermo Scientific Sensititre Vizion. MICs of the antimicrobial agents were interpreted according to the Clinical and Laboratory Standards Institute (CLSI) documents M100 34th edition. The WHONET 2024 software recommended by the WHO was used to input antimicrobial susceptibility testing data and conduct sensitivity analysis. The multiple-drug resistance (MDR) phenotype was defined as being resistant to three or more different classes of antimicrobial agents. *S. pneumoniae* ATCC 49619 was used as a quality control strain.

### Whole-genome sequencing and assembly

The total DNA of *S. pneumoniae* strains was extracted using FastPure Bacterial DNA Isolation Mini Kit (Vazyme, Nanjing, China). Proteinase K and RNAse used in the laboratory are both from Vazyme. DNA purity quotient was tested by using the spectrophotometer NanoDrop^™^ 2000 (Thermo Fisher Scientific, Waltham, MA, USA). Purified DNA was simultaneously fragmented and tagged with adapters by using the TruePrep^™^ DNA Library Prep Kit V2 for Illumina (Vazyme, Nanjing, China). Draft genome sequencing was performed on the Illumina platform NextSeq2000 with 2×150 bp paired-end reads. Raw reads were quality-controlled by removing adapter contamination, reads with >10% ambiguous bases (N), reads with >10% low-quality bases (Phred score ≤Q20) and reads shorter than 25 bp after trimming. Clean reads were assembled using SOAPdenovo v2.04 [15] for draft genome construction. Additionally, SPAdes v3.10.0 [16] and ABySS v4.2.1 [17] were used for comparative assembly. The resulting assemblies were integrated using CISA v1.3 [18] with default parameters to generate the final draft genome.

### Investigation of whole-genome characterization of *S. pneumoniae*

Average nucleotide identity (ANI) (https://pubmlst.org/bigsdb?db=pubmlst_rmlst_seqdef_kiosk) was used to identify the species based on the results of whole-genome sequencing to exclude non-pneumococcal pathogens. Qualified reads were used to predict the capsular serotypes of the strains using PneumoKIty (https://github.com/CarmenSheppard/PneumoKITy) [19]. Besides, capsular serotypes and Global Pneumococcal Sequencing Cluster (GPSC) of these genomes were also determined using the global genomic surveillance platform PathogenWatch (https://pathogen.watch/) [20]. The genome sequence of each *S. pneumoniae* isolate was submitted to the PubMLST (https://pubmlst.org) [21] *S. pneumoniae* database and aligned to multilocus sequence typing (MLST) housekeeping genes *aroE*, *gdh*, *gki*, *recP*, *spi*, *xpt* and *ddl* alleles to obtain the ST type of each strain, and the novel profiles were submitted to the pneumococcal MLST database to assign new numbers. Then the BioNumerics software was used to construct a phylogenetic tree of *S. pneumoniae* isolates based on alleles. To obtain a high-resolution phylogeny, core-genome single-nucleotide polymorphism (cgSNP) analysis was conducted with Snippy v4.6.0 (https://github.com/tseemann/snippy) using the TIGR4 genome (NC_003028) as the reference sequence, and core-genome alignment was carried out using snippy-core. The final alignment of variable sites was used to reconstruct a maximum likelihood phylogenetic tree with IQ-TREE v2.2.0 [22], and the phylogenetic tree of the isolates was visualized by iTOL v6 [23]. The whole-genome sequencing data were uploaded to the online analysis platform BacWGSTdb (http://bacdb.cn/BacWGSTdb/index.php) [24,26] to perform sequence alignment to obtain the carrying status of drug-resistance genes and virulence genes of our *S. pneumoniae* strains.

### Data processing

The data was processed with Microsoft Excel. The figures were created by BioNumerics 7.6 and RStudio 4.3.0. Statistical analysis was conducted using SPSS 22.0 software. Count data are expressed as percentages (%), and inter-group comparisons are conducted using the chi-square test or Fisher’s exact probability method; *P*<0.05 or *P*<0.001 was considered statistically significant.

## Results

### Antibiotic susceptibility of *S. pneumoniae* isolates

The susceptibilities of 105 *S*. *pneumoniae* isolates to 14 antibiotics are shown in Table 1. As we can see, in our experiment, all strains of *S. pneumoniae* were sensitive to LNZ and VAN. The sensitivity rates to ERY, CLI, TCY and SXT were low, with resistance rates as high as 92.38%, 82.86%, 86.87% and 60.95%. The resistance rates to PEN, FEP, CTX, AMX, MFX and LVX were low, while the sensitivity rates were high, at 84.73%, 74.28%, 86.87%, 70%, 96.19% and 97.14%, respectively. It is worth noting that in our antimicrobial susceptibility test (AST) of 105 strains of *S. pneumoniae*, a total of 90 strains were resistant to antibiotics of class 3 or above, with an MDR rate of 85.71%. These strains exhibited various resistance patterns, with CLI/ERY/TCY being the predominant resistance pattern. Moreover, the specific MDR combinations varied among different serotypes of *S. pneumoniae* isolates. For instance, in serotypes 19F and 23F, the most common MDR combination was CHL/CLI/ERY/TCY, while in serotype 19A, the most common MDR combination was CHL/CLI/ERY/TCY/SXT (Table S2).

**Table 1.** Resistance of 105 *S. pneumoniae* strains to 14 antibiotics, including PEN, FEP, CTX, AMX, ERY, MEM, VAN, CLI, CHL, TCY, MFX, LVX, SXT and LNZ.

Antimicrobial agent	Susceptible			Intermediate			Resistant		
	*n*	%	MIC (µg ml^−1^)	*n*	%	MIC (µg ml^−1^)	*n*	%	MIC (µg ml^−1^)
Penicillin non-meningitis	88	83.8	≤2	16	15.2	4	0	0	≥8
Penicillin meningitis	1	0.95	≤0.06	0	0	–	0	0	≥0.12
Cefepime non-meningitis	77	73.3	≤1	27	25.7	2	0	0	≥4
Cefepime meningitis	1	0.95	≤0.5	0	0	1	0	0	≥2
Cefotaxime non-meningitis	94	89.5	≤1	4	3.81	2	6	5.72	≥4
Cefotaxime meningitis	1	0.95	≤0.5	0	0	1	0	0	≥2
Amoxicillin	73	70	≤2	32	30	4	0	0	≥8
Erythromycin	6	5.72	≤0.25	2	1.9	0.5	97	92.4	≥1
Meropenem	52	49.5	≤0.25	34	32.4	0.5	19	18.1	≥1
Vancomycin	105	100	≤1	0	0	–	0		–
Clindamycin	17	16.2	≤0.25	1	0.95	0.5	87	82.9	≥1
Chloramphenicol	56	53.3	≤4	0	0		49	46.7	≥8
Tetracycline	13	12.4	≤1	1	0.95	2	91	86.7	≥4
Moxifloxacin	101	96.2	≤1	4	3.81	2	0	0	≥4
Levofloxacin	102	97.1	≤2	3	2.86	4	0	0	≥8
Trimethoprim/sulfamethoxazole	25	23.8	≤0.5/9.5	16	15.2	1/19–2/38	64	61	≥4/76
Linezolid	105	100	≤2	0	0	–	0	0	–

MIC, Minimum Inhibitory Concentration.

The results of AST (including resistant and intermediate) of *S. pneumoniae* strains isolated from patients of different ages are shown in Table 2. The resistance rate of strains isolated from the children’s group and the elderly group to trimethoprim/sulfamethoxazole was higher than that of the adult group, and the difference was statistically significant (*χ^2^=*212.143, *P*=0.002). There was no statistically significant difference in insensitivity to other antibiotics among the three age groups (*P*>0.05).

**Table 2.** Insensitivity of 105 strains of *S. pneumoniae* isolated from patients of different ages *P*<0.05 was considered statistically significant.

Antimicrobial agent	Children		Adult		Elderly		*X^2^*	*P*
	*n*	%	*n*	%	*n*	%		
Penicillin	0	0.00	0	0.00	0	0.00	na	na
Cefepime	0	0.00	0	0.00	0	0.00	na	na
Cefotaxime	4	5.48	0	0.00	2	9.09	1.079	0.583
Amoxicillin	0	0.00	0	0.00	0	0.00	na	na
Erythromycin	69	94.52	8	80.00	20	90.91	2.72	0.257
Meropenem	15	20.55	0	0.00	4	18.18	2.506	0.286
Vancomycin	0	0.00	0	0.00	0	0.00	na	na
Clindamycin	59	80.82	9	90.00	19	86.36	0.763	0.683
Chloramphenicol	31	42.47	4	40.00	14	63.64	3.242	0.198
Tetracycline	64	87.67	7	70.00	20	90.91	2.81	0.245
Moxifloxacin	0	0.00	0	0.00	0	0.00	na	na
Levofloxacin	0	0.00	0	0.00	0	0.00	na	na
Trimethoprim/sulfamethoxazole	49	67.12	1	10.00	14	63.64	12.143	0.002
Linezolid	0	0.00	0	0.00	0	0.00	na	na

### Serotype distribution and vaccine coverage rates

**Fig. 1.**
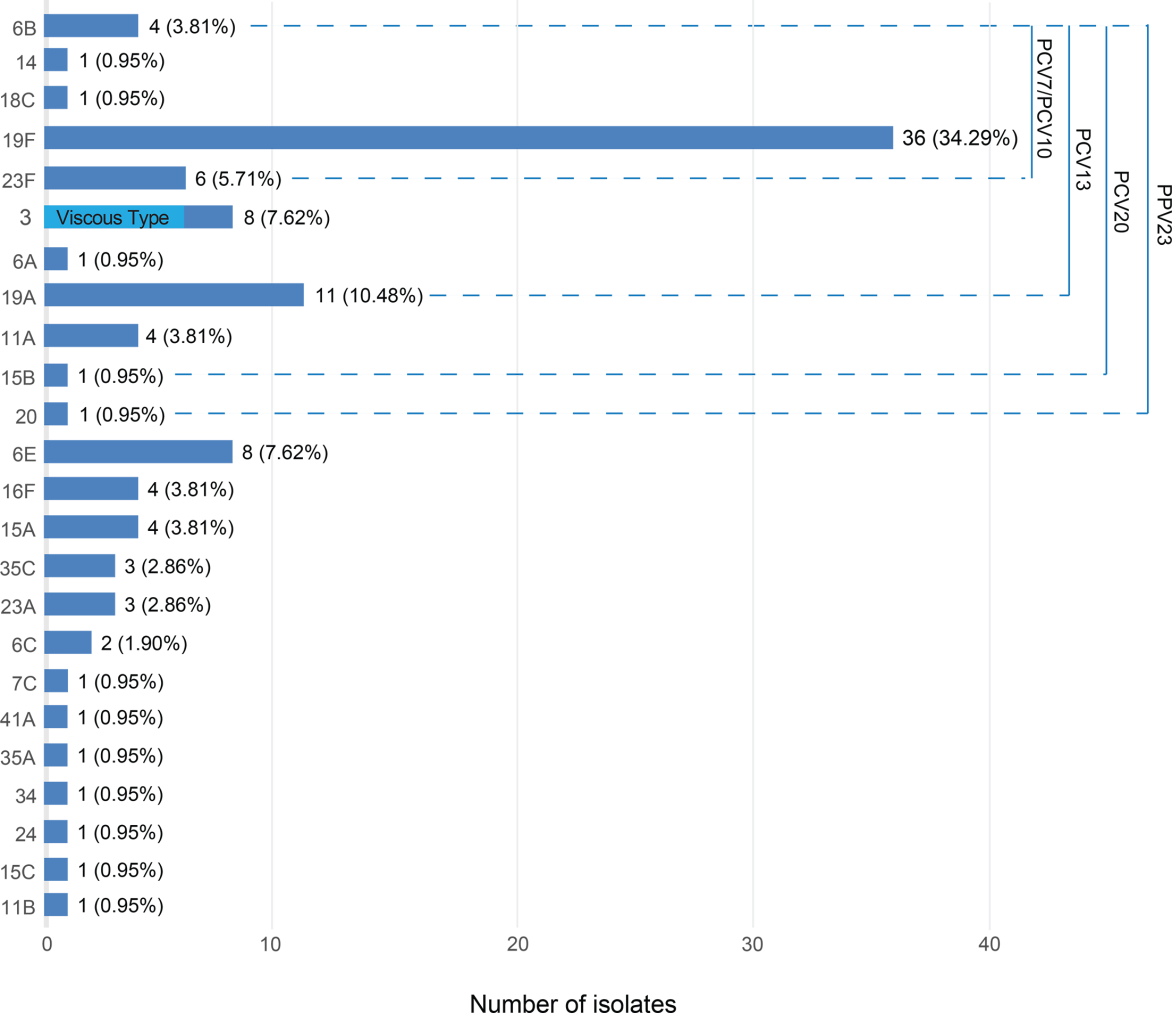
Serotype distribution of 105 *S. pneumoniae* isolates: the *x*-axis indicates the number of strains, and the *y*-axis denotes the serotypes. The serotype coverage rates for the PCV7 and PCV10 were both 45.71%. The PCV13 achieved a coverage of 64.76%. The PCV20 covered 69.52% of the isolates, while the PPV23 reached a coverage of 70.48%.

A total of 24 serotypes were involved in 105 strains of *S. pneumoniae* in this study; the main serotypes were 19F (34.29%), 19A (10.48%), 3 (7.62%) and 6E (7.62%). The serotype coverage rates for 7- and 10-valent pneumococcal conjugate vaccines (PCV7 and PCV10) were both 45.71%. There were eight serotypes and 68 isolates (64.76%) were covered by the 13-valent pneumococcal conjugate vaccine (PCV13). The 20-valent pneumococcal conjugate vaccine (PCV20) covered ten serotypes, representing 73 isolates (69.52%), whereas the 23-valent pneumococcal polysaccharide vaccine (PPV23) covered 11 serotypes, encompassing 74 isolates (70.48%). Besides, there were six cases of mucoid *S. pneumoniae* strains in our experiment, all of which belonged to serotype 3 (Fig. 1).

### Genotyping characteristics of *S. pneumoniae* strains

**Fig. 2.**
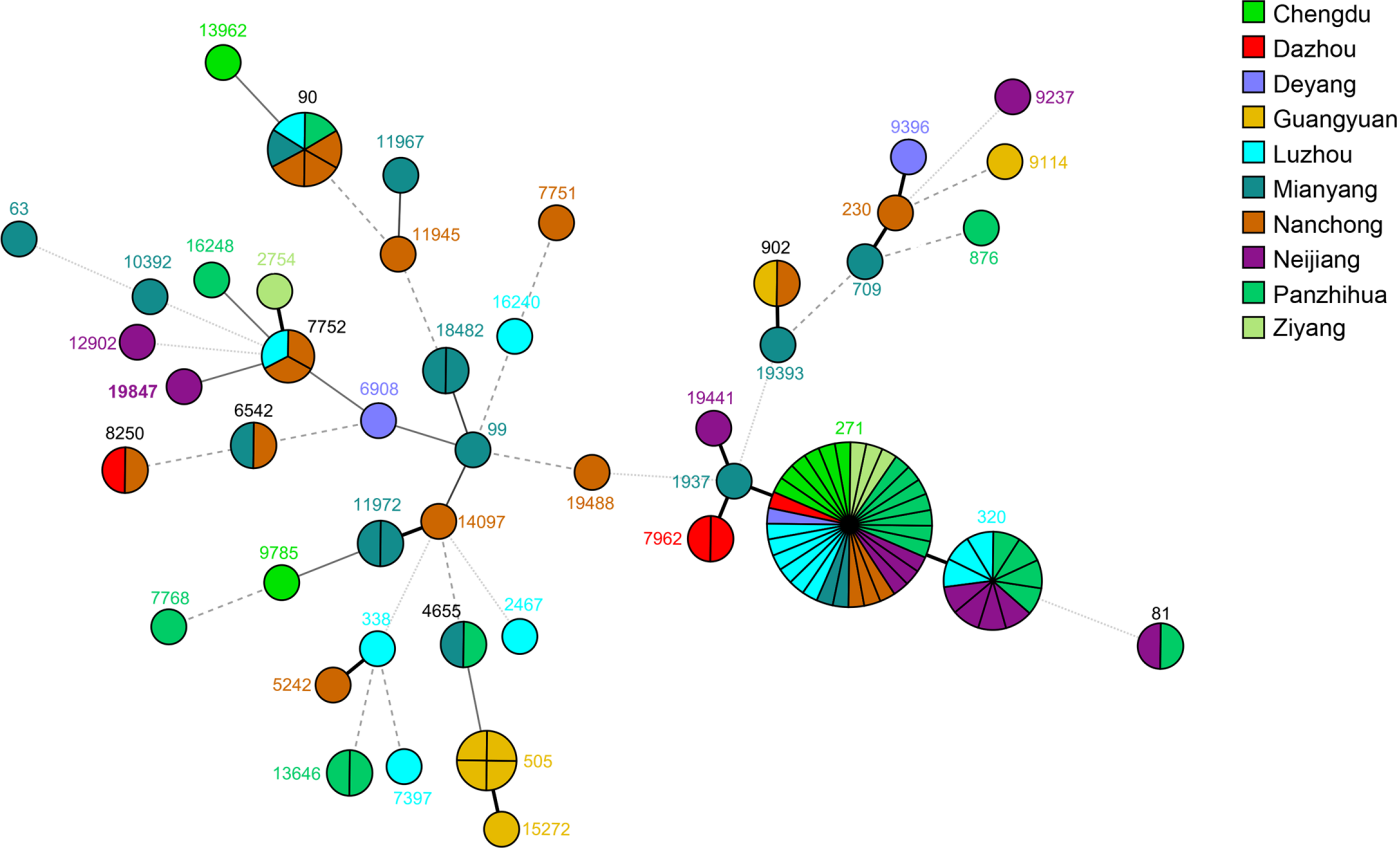
STs of 105 *S. pneumoniae* strains. Different colours represent different cities. Each circle represents the corresponding ST type. ST19847 is the newly discovered ST type in this study. The black thick solid line indicates that two STs share at least six alleles and are assigned to the same CC.

Using ANI analysis, we confirmed that all 105 isolates were *S. pneumoniae*; the quality control metrics of the 105 *S*. *pneumoniae* genomes were provided in Table S3, and all strains were qualified. Subsequently, these 105 *S*. *pneumoniae* isolates were further molecularly typed. One novel sequence type (ST) of *S. pneumoniae* was found, named ST19847. As we can see from Fig. 2, 45 different STs were classified, among which ST271 was the dominant type with 32 strains (30.48%), followed by ST320 type with 11 strains (10.48%), and the remaining 62 strains belonged to 43 different ST types. The black solid lines in this figure indicate that each MLST type has at least four or more identical alleles. And the MLST types with six or more identical alleles were classified as the same clonal complexes (CCs) in our experiment (with a black thick solid line). It can be seen that there are different genotypes in different cities, but they are also interrelated with each other. Eighteen STs were divided into seven CCs, named CC230 (ST230, ST709, ST9396), CC271 (ST271, ST320, ST1937, ST7962, ST19441), CC338 (ST338, ST5242), CC505 (ST505, ST15272), CC902 (ST902, ST19393), CC7752 (ST7752, ST2754) and CC11972 (ST11972, ST14097), representing 63.81% of all isolates (Table S4); the rest 38 STs were ungrouped. CC271 was the most prevalent clonal complex, which accounted for 44.76% (*n*=47) of the strains, and its serotype belonged to 19F (*n*=36) and 19A (*n*=11) (Table 3).

**Table 3.** Distribution of serotypes and STs in different CCs

CC	ST	Serotype	*n*	%
230	9396, 230, 709	23A, 23F, 24	3	2.86
271	271, 320, 1937, 7962, 19441	19A, 19F	47	44.76
338	338, 5242	23A	2	1.9
505	505, 15272	3	5	4.76
902	902, 19393	6B	3	2.86
7752	7752, 2754	11B, 35C	4	3.81
11972	11972, 14097	15A	3	2.86

**Fig. 3.**
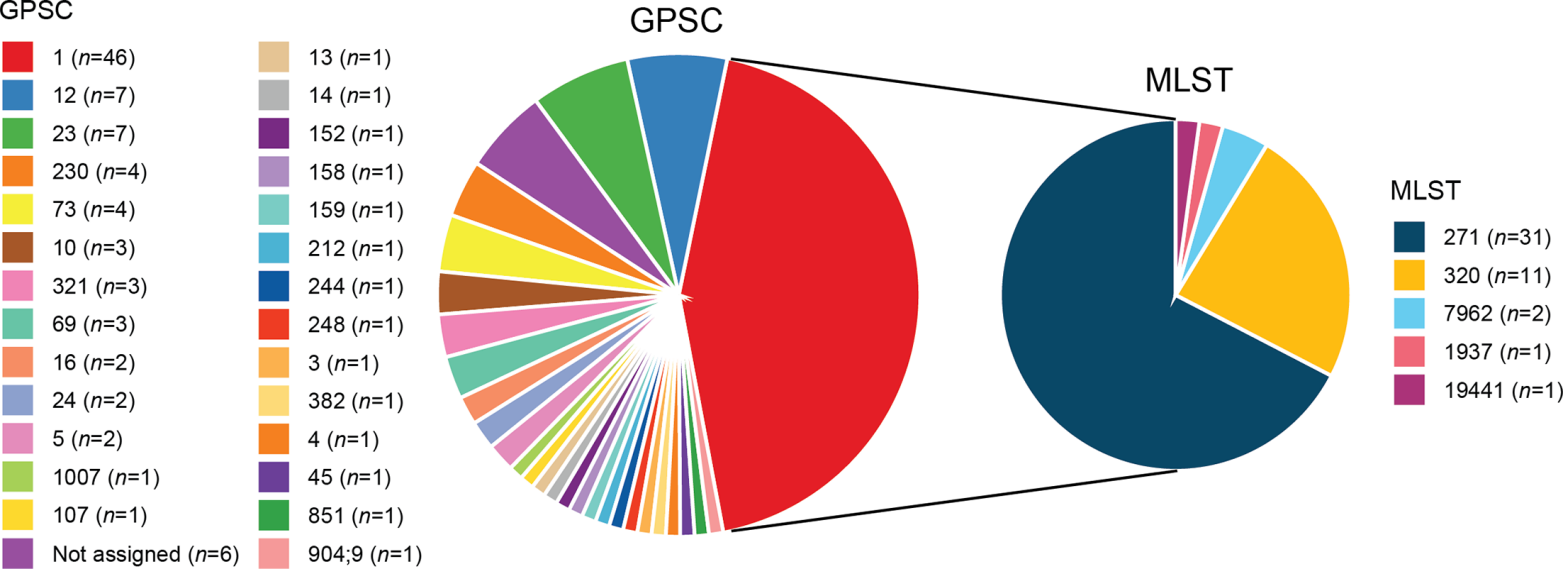
GPSCs of 105 *S. pneumoniae* strains. In the left pie chart, different colours represent different GPSCs; in the right pie chart, different colours denote the distinct MLST types within GPSC1.

Our 105 *S*. *pneumoniae* strains belonged to 27 GPSCs (6 strains were not assigned); the most prevalent GPSC was GPSC1 (Fig. 3). GPSC1 contained 46 strains (35 strains of serotype 19F and 11 strains of serotype 19A), with MLST types including ST271 (*n*=31), ST320 (*n*=11), ST7962 (*n*=2), ST1937 (*n*=1) and ST19441 (*n*=1). GPSC3 contained seven strains, all of which were serotype 3, with MLST types including ST505 (*n*=4), ST4655 (*n*=2) and ST15272 (*n*=1). GPSC23 contained seven strains, all of which were serotype 6E, with MLST types including ST90 (*n*=6) and ST13962 (*n*=1) (Table S4).

**Fig. 4.**
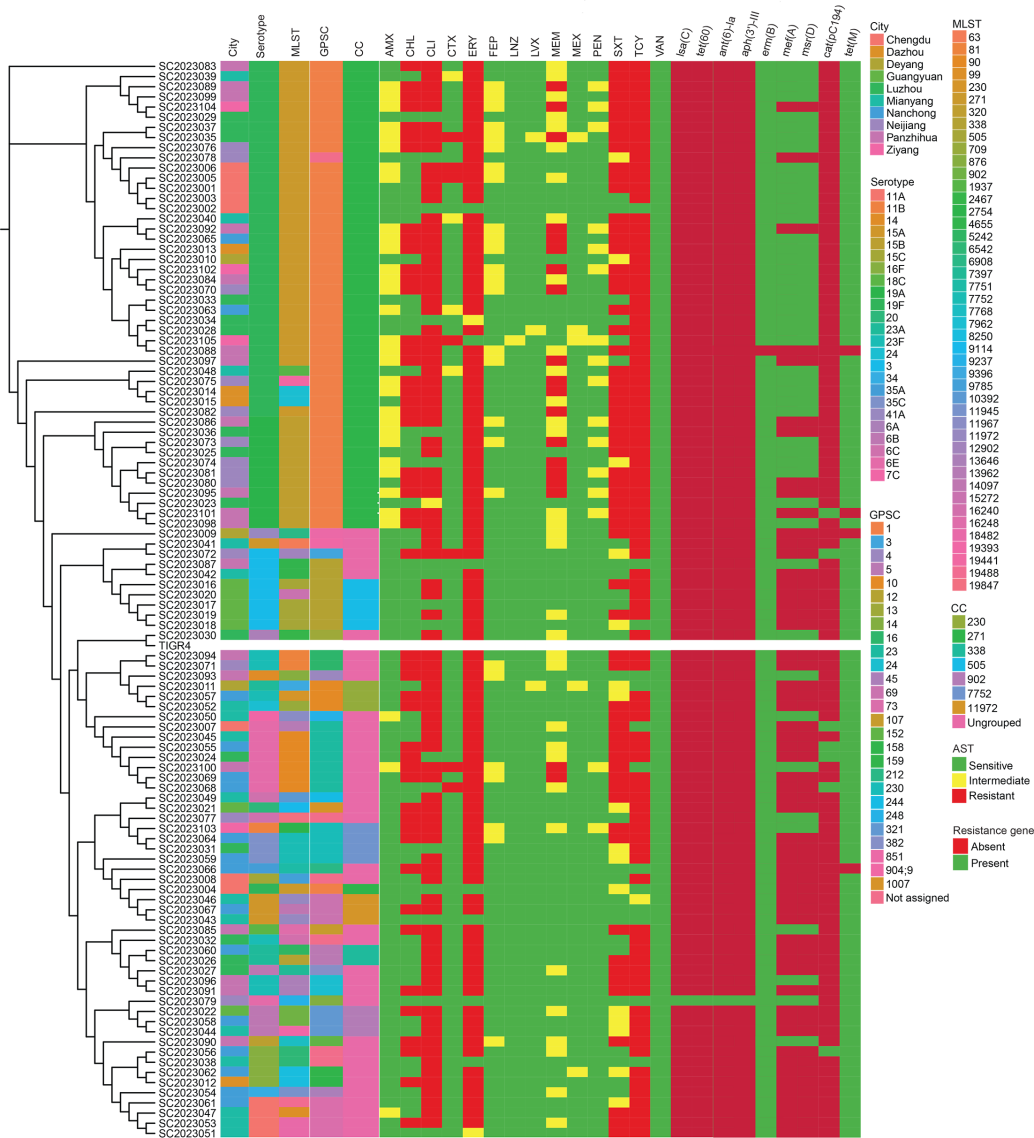
cgSNP-based phylogenetic tree of the 105 *S. pneumoniae* isolates. The heatmap displays the city of isolation, molecular characteristics (serotype, MLST, GPSC and CC), antimicrobial resistance (AMR) profiles and resistance gene (ARG) carriage. The colour of each cell in the heatmap indicated a distinct meaning. City: different colours represent different cities. Molecular characteristics: different colours represent different types (serotype, MLST, GPSC and CC). Antimicrobial resistance (AMR): green, susceptible; yellow, intermediate; red, resistant. Resistance genes (ARGs): red, absent; green, present.

As shown in Fig. 4, CC271 constituted the largest clade, encompassing multiple sequence types such as ST271 and ST320 and corresponding predominantly to serotypes 19F and 19A. These isolates formed a tight cluster, indicating high genetic relatedness. Within CC271, the carriage rate of drug resistance genes was high, and the AST profiles were mostly multidrug-resistant, generating a distinct ‘resistance cluster’ on the tree. Isolates from different cities exhibited a certain degree of geographical clustering, but given the small and uneven sample size, we cannot robustly infer transmission patterns. Conversely, some STs were detected in several cities, suggesting inter-regional spread of these clones.

### Drug resistance genes and virulence genes of *S. pneumoniae*

A total of 9 drug resistance genes were detected in 105 *S*. *pneumoniae* strains, including macrolides [*erm(B*), *mef(A*), *msrD*], tetracyclines [*lsa(C*), *tetA(60*), *tet(M*)], amphenicols [*cat (pC194*)] and aminoglycoside [*aph(3′)-III*, *ant(6)-Ia*] resistance genes (Table 4). We can find that 104 strains (99.05%) carried *erm(B*), 47 strains carried both *mef(A*) and *msr(D*) and 101 strains (96.19%) carried the *tet(M*). Based on our drug susceptibility phenotype experiments, it was found that among the 96 strains exhibiting ERY resistance, 45.83% carried both *erm(B*) and *mef(A)+msr(D*) genes, while the rest carried *erm(B*) alone. Among the 88 strains exhibiting TCY resistance, all of them carried *tet(M*) (Table S2).

**Table 4.** Characteristics of resistance genes in 105 *S. pneumoniae* isolates *P*<0.001 was considered statistically significant.

Resistance gene		Total	%	Serotype										*P*	GPSC								*P*
				19F	%	19A	%	3	%	6E	%	Others	%		GPSC1	%	GPSC12	%	GPSC23	%	Others	%	
Macrolides	*erm(B*)	104	99.05	35	97.22	11	100.00	8	100.00	8	100.00	42	100.00	0.600	45	97.83	7	100.00	7	100.00	45	100.00	1.000
	*mef(A*)	47	44.76	30	83.33	6	54.55	1	12.50	2	25.00	8	19.05	**<0.001**	36	78.26	1	14.29	1	14.29	9	20.00	**<0.001**
	*msr(D*)	47	44.76	30	83.33	6	54.55	1	12.50	2	25.00	8	19.05	**<0.001**	36	78.26	1	14.29	1	14.29	9	20.00	**<0.001**
Tetracyclines	*lsa(C*)	1	0.95	0	0.00	1	9.09	0	0.00	0	0.00	0	0.00	0.257	0	0.00	0	0.00	0	0.00	1	2.22	0.562
	*tetA(60*)	1	0.95	0	0.00	1	9.09	0	0.00	0	0.00	0	0.00	0.257	0	0.00	0	0.00	0	0.00	1	2.22	0.562
	*tet(M*)	101	96.19	35	97.22	11	100.00	8	100.00	7	87.50	40	95.24	0.605	44	95.65	7	100.00	7	100.00	43	95.56	1.000
Amphenicols	*cat(pC194*)	7	6.67	0	0.00	1	9.09	1	12.50	4	50.00	1	2.38	**<0.001**	1	2.17	0	0.00	4	57.14	2	4.44	0.001
Aminoglycosides	*aph(3')-III*	1	0.95	0	0.00	0	0.00	0	0.00	1	12.50	0	0.00	0.152	0	0.00	0	0.00	0	0.00	1	2.22	0.562
	*ant(6)-Ia*	1	0.95	0	0.00	0	0.00	0	0.00	1	12.50	0	0.00	0.152	0	0.00	0	0.00	0	0.00	1	2.22	0.562

Besides, our 105 *S*. *pneumoniae* strains identified five classes of virulence factors, including adhesion genes (*pspC*, *cbpG*, *pce*, *pavA*, *pfbA*, *pitA, pitB*, *sipA*, *rrgA*, *rrgB*, *rrgC*, *srtG1*, *srtG2*, *srtC-1*, *srtC-2* and *srtC-3*), exotoxin gene (*ply*), ectoenzyme genes (*cbpD*, *lytA*, *lytB*, *lytC*, *hysA*, *nanA* and *nanB*), immunomodulatory factors (*cps4A*) and nutritional metabolic factors (*cps4B*, *cps4D*, *zmpC* and *psaA*) (Table 5). Among these virulence genes, most of the strains carried *pce* (80.0%), *pavA* (99.1%), *ply* (99.1%), *cbpD* (91.4%), *lytA* (98.1%), *lytC* (94.3%), *hysA* (95.2%), *nanB* (88.6%), *cps4A* (98.1%), *cps4B* (97.1%) and *psaA* (99.1%). In addition, more than half of the strains carried *pfbA* (58.1%) and *nanA* (77.1%).

**Table 5.** Characteristics of virulence genes in 105 *S. pneumoniae* isolates *P*<0.001 was considered statistically significant.

Virulence gene		Total	%	Serotype										*P*	GPSCs								*P*
				19F	%	19A	%	3	%	6E	%	Others	%		GPSC1	%	GPSC12	%	GPSC23	%	Others	%	
Adhesion genes	*pspC*	2	1.90	0	0.00	0	0.00	0	0.00	0	0.00	2	4.76	0.723	0	0.00	0	0.00	0	0.00	2	4.44	0.431
	*cbpG*	35	33.33	1	2.78	2	18.18	1	12.50	6	75.00	25	59.52	**<0.001**	3	6.52	0	0.00	6	85.71	26	57.78	**<0.001**
	*pce*	84	80.00	32	88.89	10	90.91	1	12.50	8	100.00	33	78.57	**<0.001**	41	89.13	1	14.29	7	100.00	35	77.78	**<0.001**
	*pavA*	104	99.05	36	100.00	10	90.91	8	100.00	8	100.00	42	100.00	0.257	45	97.83	7	100.00	7	100.00	45	100.00	1.000
	*pfbA*	61	58.10	8	22.22	4	36.36	7	87.50	6	75.00	36	85.71	**<0.001**	11	23.91	6	85.71	6	85.71	38	84.44	**<0.001**
	*pitA*	45	42.86	30	83.33	6	54.55	1	12.50	2	25.00	6	14.29	**<0.001**	36	78.26	1	14.29	1	14.29	7	15.56	**<0.001**
	*pitB*	45	42.86	30	83.33	6	54.55	1	12.50	2	25.00	6	14.29	**<0.001**	36	78.26	1	14.29	1	14.29	7	15.56	**<0.001**
	*sipA*	45	42.86	30	83.33	6	54.55	1	12.50	2	25.00	6	14.29	**<0.001**	36	78.26	1	14.29	1	14.29	7	15.56	**<0.001**
	*rrgA*	45	42.86	29	80.56	6	54.55	1	12.50	2	25.00	7	16.67	**<0.001**	35	76.09	1	14.29	1	14.29	8	17.78	**<0.001**
	*rrgB*	42	40.00	29	80.56	3	27.27	1	12.50	2	25.00	7	16.67	**<0.001**	32	69.57	1	14.29	1	14.29	8	17.78	**<0.001**
	*rrgC*	52	49.52	29	80.56	7	63.64	1	12.50	8	100.00	7	16.67	**<0.001**	36	78.26	1	14.29	7	100.00	8	17.78	**<0.001**
	*srtG1*	45	42.86	30	83.33	6	54.55	1	12.50	2	25.00	6	14.29	**<0.001**	36	78.26	1	14.29	1	14.29	7	15.56	**<0.001**
	*srtG2*	45	42.86	30	83.33	6	54.55	1	12.50	2	25.00	6	14.29	**<0.001**	36	78.26	1	14.29	1	14.29	7	15.56	**<0.001**
	*srtC-1*	52	49.52	29	80.56	7	63.64	1	12.50	8	100.00	7	16.67	**<0.001**	36	78.26	1	14.29	7	100.00	8	17.78	**<0.001**
	*srtC-2*	52	49.52	29	80.56	7	63.64	1	12.50	8	100.00	7	16.67	**<0.001**	36	78.26	1	14.29	7	100.00	8	17.78	**<0.001**
	*srtC-3*	42	40.00	29	80.56	3	27.27	1	12.50	2	25.00	7	16.67	**<0.001**	32	69.57	1	14.29	1	14.29	8	17.78	**<0.001**
Exotoxin gene	*ply*	104	99.05	36	100.00	10	90.91	8	100.00	8	100.00	42	100.00	0.257	45	97.83	7	100.00	7	100.00	45	100.00	1.000
Ectoenzyme genes	*cbpD*	96	91.43	30	83.33	9	81.82	8	100.00	8	100.00	41	97.62	0.096	38	82.61	7	100.00	7	100.00	44	97.78	0.070
	*lytA*	103	98.10	36	100.00	10	90.91	8	100.00	8	100.00	41	97.62	0.450	45	97.83	7	100.00	7	100.00	44	97.78	1.000
	*lytB*	28	26.67	5	13.89	2	18.18	4	50.00	0	0.00	17	40.48	0.011	6	13.04	3	42.86	0	0.00	19	42.22	0.002
	*lytC*	99	94.29	36	100.00	10	90.91	3	37.50	8	100.00	42	100.00	**<0.001**	45	97.83	2	28.57	7	100.00	45	100.00	**<0.001**
	*hysA*	100	95.24	32	88.89	10	90.91	8	100.00	8	100.00	42	100.00	0.141	41	89.13	7	100.00	7	100.00	45	100.00	0.156
	*nanA*	81	77.14	34	94.44	9	81.82	8	100.00	8	100.00	22	52.38	**<0.001**	42	91.30	7	100.00	7	100.00	25	55.56	**<0.001**
	*nanB*	93	88.57	35	97.22	9	81.82	7	87.50	2	25.00	40	95.24	**<0.001**	43	93.48	6	85.71	1	14.29	43	95.56	**<0.001**
Immunomodulatory factors	*cps4A*	103	98.10	36	100.00	10	90.91	7	87.50	8	100.00	42	100.00	0.064	45	97.83	6	85.71	7	100.00	45	100.00	0.250
Nutritional metabolic factors	*cps4B*	102	97.14	36	100.00	10	90.91	6	75.00	8	100.00	42	100.00	0.007	45	97.83	6	85.71	7	100.00	44	97.78	0.428
	*cps4D*	49	46.67	26	72.22	2	18.18	0	0.00	2	25.00	19	45.24	**<0.001**	28	60.87	0	0.00	1	14.29	20	44.44	0.003
	*zmpC*	3	2.86	0	0.00	0	0.00	1	12.50	0	0.00	2	4.76	0.289	0	0.00	0	0.00	0	0.00	3	6.67	0.273
	*psaA*	104	99.05	36	100.00	10	90.91	8	100.00	8	100.00	42	100.00	0.257	45	97.83	7	100.00	7	100.00	45	100.00	1.000

The gene carriage rate varied significantly among strains of different serotypes. For macrolide resistance genes, the carriage rates of *mef(A*) and *msr(D*) in GPSC1 were significantly higher than those of other GPSCs, and the carriage rates of these two genes were highest in serotype 19F, both at 83.3% (Table 4). In GPSC1, the carriage rates of most adherence genes were significantly higher than those of other GPSCs, including *pitA*, *pitB*, *sipA*, *rrgA*, *rrgB*, *rrgC*, *srtG1*, *srtG2*, *srtC-1*, *srtC-2* and *srtC-3*. And the carriage rates of these genes in serotype 19F were also much higher than other serotypes. *pfbA* was rarely detected in serotype 19F but was prevalent in other serotypes. For exoenzymes, the carriage rate of *nanB* was lowest in GPSC23 (serotype 6E). For nutritional metabolic factors, the carriage rate of *cps4D* was lowest in serotype 3 (Table 5).

## Discussion

*S. pneumoniae*, as an important human pathogen, is the main cause of community-acquired pneumonia, meningitis, sepsis and other invasive pneumococcal diseases [2]. With the widespread use of antibiotics, the resistance of *S. pneumoniae* to multiple antibiotics has become increasingly severe, posing a significant challenge to global public health [27,29]. Our study aimed to investigate the molecular characteristics and antibiotic resistance of 105 strains of *S. pneumoniae* in Sichuan Province in 2023 through whole-genome sequencing and antibiotic susceptibility tests, in order to provide a scientific basis and data support for the prevention and control of *S. pneumoniae* outbreaks. We utilized whole-genome sequencing to comprehensively reveal the genomic information of *S. pneumoniae*, including typing, resistance genes and virulence factors, providing a basis for accurate identification of strains and prediction of their transmission potential. At the same time, combined with antibiotic susceptibility tests, the sensitivity of different bacterial strains to commonly used antibiotics is clarified, guiding for rational clinical drug use. The research results will help optimize the prevention and control strategies of *S. pneumoniae* in Sichuan Province, improve public health response capabilities and provide important references for reducing the incidence and drug resistance of *S. pneumoniae* infections.

In recent years, the insensitivity rate of *S. pneumoniae* strains to antibiotics in China has remained at a high level. Fang Chao *et al*. found that the resistance rates of pneumococcal isolates from children in China to erythromycin, clindamycin and tetracycline were as high as 97.9%, 95.9% and 93.2%, respectively [30]. In our antimicrobial susceptibility testing of the 105 strains of pneumococcus this time, we found that the results were similar to the previously reported drug resistance of *S. pneumoniae*. The strains showed high resistance rates to erythromycin and tetracycline, but high sensitivity to levofloxacin and moxifloxacin. Besides, our drug resistance rates (ERY 97.1%, TET 88.6%) are at the upper end of the 2023 GPS-Asia summary (ERY 91.3–97.2%; TET 83.1–90.4%), but far above the contemporary European medians (ERY 24.6%, TET 18.7%), confirming East Asia as a global hot-spot (https://www.pneumogen.net/gps/reports). No *S. pneumoniae* strains resistant to vancomycin and linezolid were found [31]. There are multiple MDR patterns in *S. pneumoniae* strains, with 85.71% of strains simultaneously resistant to three or more antibiotics. It is worth noting that our study revealed a significantly higher prevalence of resistance to trimethoprim/sulfamethoxazole among children and the elderly than among adults, most likely driven by frequent empirical use of this agent in these age groups. Additionally, factors such as weakened immune function in these populations may also contribute, but we did not investigate this directly. In addition, we also observed the distribution patterns of the resistance genes in these strains. Although 104 strains contained the macrolide *erm(B*) gene, only 96 strains exhibited erythromycin resistance, among which 44 strains simultaneously contained the *erm(B)+mef(A)+msr(D*) genes. The *erm(B*) gene is widely present in *S. pneumoniae* and other bacteria worldwide and is one of the main genes causing resistance to macrolides. In some regions, the carrier rate of the *erm(B*) gene is very high. For example, in Hebei Province, the carrier rate of *erm(B*) in *S. pneumoniae* is as high as 96.00%. Bacteria carrying the *erm(B*) gene may be resistant to multiple antibiotics at the same time, which increases the difficulty of treatment and may lead to the emergence of multidrug-resistant bacteria [32]. Some *S. pneumoniae* strains may simultaneously carry the *mef(A*) and *erm(B*) genes, showing resistance to macrolide and lincosamide antibiotics (MLSB type), and the *msr(D*) gene can be cotranscribed with *mef(A*), leading to bacterial resistance to antibiotics [33]. Although 101 strains were detected with the *tet(M*) gene, only 88 strains exhibited tetracycline resistance. In our study, some isolates carried the macrolide-resistance gene *erm(B*) (7.69%), or the tetracycline-resistance gene *tet(M*) (12.87%), yet remained phenotypically susceptible, figures almost identical to those reported in the GPS global survey – 9.4% for *erm(B*) and 10.8% for *tet(M*) (https://www.pneumogen.net/gps/reports). This discrepancy is most likely explained by (i) incomplete or non-functional genes and/or (ii) expression levels that do not reach the clinical breakpoint [34]. It can be seen from this that the presence of these resistance genes does not directly lead to the emergence of a resistant phenotype in the strains, but may be related to the development of antibiotic resistance in *S. pneumoniae*. At the same time, these data can remind us to use antibiotics more rationally in clinical treatment to reduce the further development of resistant strains. It also emphasizes the importance of continuously monitoring the changing trends of pneumococcus drug resistance to provide timely guidance for clinical treatment.

We identified a total of 29 virulence genes in 5 categories. Among them, the detection rates of adhesion gene *pavA*; exotoxin gene *ply*; ectoenzyme genes *lytA*, *hysA* and *lytC*; immunomodulatory genes *cps4A* and *cps4B*; and nutritional metabolic gene *psaA* were relatively high. This is similar to the reports from Hebei Province and Shanghai, but contrary to the results reported in Ningbo [32,35, 36]. This suggests that the detection rate of virulence genes varies between published studies from different locations in China, which may reflect differences in sampling, patient populations or local pneumococcal populations. In pneumococcal isolates from China, *lytA*, *ply*, *hysA* and *nanA* are the most common virulence genes, with positive rates ranging from 95 to 100% [37]. The *pavA* and *psaA* genes are significantly associated with the occurrence of pneumococcal bacteraemia and meningitis [38,39]. Most of our isolates carried *lytA*, *ply*, *psaA*, *nanA*, *pavA* and *piaA*, which is consistent with reports from other cities in China [39]. Previous studies have shown that lytA, owing to its autolytic activity and high conservation (>98% amino-acid identity across pneumococcal genomes), has recently been incorporated into a multi-epitope chimeric vaccine (in tandem with pspA-derived epitopes) that conferred 90% survival in a mouse septicaemia challenge model [40]. Immunization with the ply variant plyD1 protects mice against lethal *S. pneumoniae* challenge and lung injury [41]. Mutations in psaA result in global defects in growth, virulence, adherence and oxidative-stress response [42]. PavA and piaA are often covalently linked as a bivalent conjugate to CRM197 or adsorbed onto Al(OH)₃, which significantly enhances cross-protection in mice against pneumococcal serotypes such as 6B and 19F [43]. Based on the current research findings, we considered that these virulence factors, *lytA*, *ply*, *psaA*, *nanA*, *pavA* and *piaA*, may become potential candidates for future pneumococcal vaccines.

A total of 53 serotypes of *S. pneumoniae* have been reported in China, with the most common serotype being 19F [44]. In our current experiment, the most common serotypes were 19F (36 strains, 34.29%) and 19A (11 strains, 10.48%). For the 19F serotype strains, there were 4 MLST types, among which ST271 was the predominant MLST type (32 strains, 88.89%). For the 19A serotype strains, there was only one ST320 type, which is similar to the report from Zhongjiang County, Sichuan Province, previously [45]. The CC271 is one of the most important clonal complexes of *S. pneumoniae* in China at present, and the two dominant clones are 19F ST271-B and 19A ST320 [46]. Similarly, in our experiment, CC271 was the most prevalent CC. GPSC1, which was the most frequent in our study, was associated with serotypes 19F and 19A, and the isolates were mostly multidrug-resistant. The cgSNP phylogeny further identified CC271 as the dominant multidrug-resistant clonal complex of *S. pneumoniae* in Sichuan, predominantly associated with serotypes 19F and 19A and exhibiting wide dissemination. Its genetic structure was closely linked to city of origin, serotype and antimicrobial resistance, underscoring that vaccine selection and antibiotic stewardship should be grounded in local molecular epidemiology. Moreover, strains carrying novel sequence types and non-vaccine serotypes displayed high genetic diversity, necessitating continuous surveillance of their evolutionary trajectories. Notably, SC2023083 formed a separate branch in the phylogenetic tree, suggesting that it may have been introduced from outside the local region. Its potential for future spread should therefore be monitored continuously.

Studies have confirmed that pneumococcal vaccination can significantly reduce pneumonia caused by vaccine-covered serotypes (VT). Understanding the distribution of pneumococcal serotypes is a key factor in formulating vaccination strategies. In China, PCV13 was launched in June 2017 and quickly replaced PCV7 as the main pneumococcal conjugate vaccine for children due to its broader serotype coverage and better cost-effectiveness. The pneumococcal vaccine not only can reduce the risk and severity of individual illness, but also can reduce the use of antibiotics, and it can reduce the carrier rate of pneumococcal bacteria, effectively reducing the spread of the disease in the population. However, according to the data reported by the Chinese Center for Disease Control and Prevention, although the vaccination rate of the pneumococcal vaccine in Sichuan Province increased year by year from 2019 to 2021, by 2021, the full-course vaccination completion rate in Sichuan Province was still only 16.45%, far lower than that of the eastern regions during the same period [47]. In this study, the vaccine-covered serotypes of PCV7 and PCV10 accounted for 45.71%, those of PCV13 were 64.76%, PCV20 covered 69.52% and PPV23 covered 70.48%. These data illustrated that pneumococcal vaccination not only reduces individual risk of disease and its severity and curtails antibiotic use, but also reduces the carriage rate of pneumococcus, effectively reducing the spread of the disease among the population. However, PPV23 is currently licensed in China only for older adults or individuals with specific high-risk conditions [7]. We should therefore implement measures to promote pneumococcal vaccine uptake across all target groups and accelerate the introduction of PCV20.

Overall, our study provides valuable insights into the serotype, molecular characteristics and antibiotic susceptibility of *S. pneumoniae* strains in Sichuan Province. The most common strains in this study were 19F, 19A, 3 and 6E. The most prevalent GPSCs were GPSC1, GPSC12 and GPSC23. The most prevalent STs were ST271 and ST320. The most important CC was CC271. All strains were sensitive to VAN and LNZ, but highly resistant to ERY, CLI, TCY and SXT, with a concerning MDR of up to 85.71%. These data highlight the importance of the appropriate use of antibiotics and emphasize the necessity of monitoring pneumococcal epidemiology. In our study, PCV13 covered 64.76% of the isolates, PCV20 covered 69.52% and PPV23 covered 70.48%. Given that only PCV13 and PPV23 are currently licensed in China and the persistently high rate of antibiotic non-susceptibility, efforts should be made to consolidate the implementation of PCV13 and PPV23 while actively facilitating the clinical introduction of PCV20. This combined strategy will improve the prevention and control of pneumococcal infections in Sichuan Province. Continuous monitoring of the molecular characteristics and antibiotic resistance of *S. pneumoniae* is crucial for controlling and preventing pneumococcal infections.

The *S. pneumoniae* isolates collected in this study originated from ten cities across Sichuan Province and covered patients of all age groups, providing broad but still limited regional coverage. Whole-genome sequencing was employed to simultaneously determine serotype, MLST, resistance and virulence genes and GPS clonal complexes, thereby avoiding the biases associated with conventional PCR-based typing. Besides, CLSI-compliant antimicrobial susceptibility testing was performed in parallel, allowing us to systematically document the local *erm(B*)/*tet(M*) genotype–phenotype discordance and to benchmark our findings against the global GPS dataset, which enhances international comparability. Forward-looking estimates of PCV20 coverage were also generated to provide direct evidence for future vaccine introduction. However, several limitations should be acknowledged. First, the 105 clinical isolates were collected within a single calendar year, and the overall sample size is relatively small, which may not capture seasonal or year-to-year fluctuations. Second, only a small subset were invasive isolates, limiting the statistical power to accurately estimate serotype distribution, resistance rates or virulence-gene frequencies among invasive strains. Third, the mechanisms underlying genotype–phenotype discrepancies were not explored by transcriptional or enzymatic assays, leaving the biological basis only partially clarified. Fourth, the number of isolates varied significantly across cities, resulting in limited geographical representativeness. Therefore, the scope of surveillance and the sample size should be expanded in future continuous monitoring. Finally, the additional serotypes included in PCV20 are currently rare in this region, so the real-world effectiveness of PCV20 will need to be reassessed after the vaccine is licensed and deployed. All in all, continuous surveillance of *S. pneumoniae* in our region is, therefore, essential.

## Supplementary material

10.1099/jmm.0.002107 Uncited Supplementary Material 1.

## Abbreviations

AMX: amoxicillin
ANI: average nucleotide identity
AST: antimicrobial susceptibility test
CC: clonal complex
cgSNP: core-genome single-nucleotide polymorphism
CHL: chloramphenicol
CLI: clindamycin
CLSI: Clinical and Laboratory Standards Institute
CTX: cefotaxime
ERY: erythromycin
FEP: cefepime
GPSC: Global Pneumococcal Sequencing Cluster
LNZ: linezolid
LVX: levofloxacin
MDR: multidrug resistance
MEM: meropenem
MFX: moxifloxacin
MLST: multilocus sequence typing
PCV7: 7-valent pneumococcal conjugate vaccine
PCV10: 10-valent pneumococcal conjugate vaccine
PCV13: 13-valent pneumococcal conjugate vaccine
PCV20: 20-valent pneumococcal conjugate vaccine
PEN: penicillin
PPV23: 23-valent pneumococcal polysaccharide vaccine
STs: sequence types
SXT: trimethoprim/sulfamethoxazole
TCY: tetracycline
VAN: vancomycin
WHO: World Health Organization
